# Efficient Deep Learning-Based Device-Free Indoor Localization Using Passive Infrared Sensors

**DOI:** 10.3390/s25051362

**Published:** 2025-02-23

**Authors:** Sira Yongchareon, Jian Yu, Jing Ma

**Affiliations:** School of Engineering, Computer and Mathematical Sciences, Auckland University of Technology, Auckland 1010, New Zealand; jian.yu@aut.ac.nz (J.Y.); jing.ma@aut.ac.nz (J.M.)

**Keywords:** device-free indoor localization, multi-person localization, deep learning-based localization, PIRs

## Abstract

Internet of Things (IoT) technology has continuously advanced over the past decade. As a result, device-free indoor localization functions have become a crucial part of application areas such as healthcare, safety, and energy management. Passive infrared (PIR) sensors detecting changes in temperature in an environment are one of the suitable options for human localization due to their lower cost, low energy consumption, electromagnetic tolerance, and enhanced private awareness. Although existing localization methods, including machine/deep learning, have been proposed to detect multiple persons based on signal phase and amplitude, they still face challenges regarding signal quality, ambiguity, and interference caused by the complex, interleaving movements of multiple persons. This paper proposes a novel deep learning method for multi-person localization using channel separation and template-matching techniques. The approach is based on a deep CNN-LSTM architecture with ensemble models using a mean bagging technique for achieving higher localization accuracy. Our results show that the proposed method can estimate the locations of two participants simultaneously with a mean distance error of 0.55 m, and 80% of the distance errors are within 0.8 m.

## 1. Introduction

Indoor localization is a key component of many useful application areas, such as healthcare, energy management, and security management [1,2]. Localization information enables us to respond appropriately and efficiently to any important event, such as medical or security events. In general, indoor localization can be categorized into device-based and device-free localization. For device-based localization, people are required to wear wearable devices that can provide location information, such as mobile phones, smartwatches, and tracking bracelets. However, this requirement is unsuitable when people do not cooperate due to intrusiveness or when everyone cannot wear a device [3]. These issues give rise to device-free localization approaches where persons can be located or localized without requiring them to wear or hold any device [4,5]. In addition, Internet of Things (IoT) technology has vastly improved over the past decade. As a result, the improvement further increases the capability of device-free localization in areas such as accuracy, coverage distance, and sensitivity. Various technologies can be employed to implement device-free localization. Many works commonly use images and radio frequency (RF). However, there are a few drawbacks, including privacy issues, energy consumption, and hardware costs [6,7].

Passive infrared (PIR) sensors have a low unit price and are easily acquired off the shelf. They can operate under any lighting condition, consume less energy, and are unaffected by electromagnetic interference [8,9]. On the other hand, PIR sensors cannot detect through walls, and their detection sensitivity is affected by a rise and fall in background temperature. Despite these drawbacks, PIR sensors have been successfully employed as a low-cost indoor localization solution [10]. Existing works require either a dense deployment of PIR sensors [11,12] or sophisticated PIR nodes [6,13], which are challenging to implement in real life and do not achieve good results in multi-person scenarios. Therefore, this study aims to leverage analogue output features to improve localization for multiple persons. Compared with binary outputs, PIR’s analogue outputs are feature-rich. In utilizing analogue outputs, the number of deployed PIR sensors would be reduced significantly [14].

Deep learning approaches have emerged over the past years and have been adopted successfully in human sensing (e.g., [15,16,17]) as deep learning overcomes a key challenge in traditional machine learning, such as complex feature extraction [18]. However, some challenges need to be addressed to realize multi-person localization with a deep learning approach. Data collection for multiple persons can be time-consuming, and it is impossible to collect data for a deep learning model that can cope with every walking scenario of multiple persons. Multiple persons can introduce challenges regarding signal ambiguity and interference caused by complex, interleaving movements of multiple persons. Thus, unexpected patterns can occur, and a trained deep learning model cannot recognize them. To address the above challenges, a deep learning model based on template matching and mean bagging is proposed to achieve accurate multi-person localization.

The main contributions of this study are summarized as follows:

- A robust, single-node PIR-based indoor localization approach for multiple persons based on a channel separation method was developed to generate inputs for multiple persons by summating an existing dataset.
- Ensemble learning was used to handle the ambiguity of signals caused by the presence of multiple persons.
- A series of experiments were conducted to evaluate the effectiveness of the approach, yielding promising results with a mean distance error of 0.55 m, and 80% of the errors were within 0.8 m.

Table 1 presents an overall comparison of existing indoor localization technologies, including our proposed method.

The rest of this paper is organized as follows. Section 2 first discusses related works on indoor localization technology. Then, Section 3 proposes a methodology for multi-person-based PIR localization. Section 4 describes the setup of experiments for both hardware and data collection. After that, Section 5 discusses the results of the experiments. Finally, a summary of the proposed work is presented in Section 6.

## 2. Related Work

Device-free indoor localization technology can be divided into two categories: non-infrared- and infrared-based. For non-infrared-based technology, radio frequency (RF) technology, including Zigbee, WIFI, RFID, UWB, and mmWave, has been employed in many works due to its coverage distance and obstacle-penetrating capability [19]. However, RF localization is susceptible to electromagnetic interference, a multipath effect that can affect the accuracy of localization [20]. In addition, hardware costs and power consumption are considerably high in some RF technologies, such as WiFi and mmWave [3]. The commonly used localization techniques for RF technology include proximity, triangulation, imaging, and fingerprinting. The proximity method can localize multiple persons at different sensors but may require dense deployment to improve localization accuracy [21,22]. The triangulation method utilizes time of arrival (TOA) and angle of arrival (AOA) to estimate the locations of multiple persons, but reflected signals interfere with each other due to the presence of multiple persons [23,24,25]. Fingerprinting techniques can localize multiple persons in different zones [26]. In addition, localizing different people in the same zone can be achieved by analyzing the impact of a single person and subtracting it from the received measurement recursively [27]. The imaging method can locate multiple persons that cause signal attenuation by obstructing radio links at different locations [18,28]. Capacitive floor technology can determine the locations of different persons who step directly on the capacitive mats at different locations. It can give an accurate result but may not be practical due to the difficulty of floor mat installation [29,30]. Acoustic or vibration can localize a person using triangulation techniques similar to RF technology and requires us to separate sources from localizing multiple targets [9,31].

For infrared-based technology, both PIRs and thermopile sensors are commonly used for localization. In general, changes in temperature on the sensing element of both sensors are converted to either analogue output or binary output through multiple steps of the thermal-to-voltage conversion. Passive infrared sensors can be acquired at a low price and low energy consumption, and they can achieve a localization error that is less than 1 m for localization purposes. Some drawbacks include low coverage distance and background temperature, which can affect their sensitivity. However, they are still suitable for indoor environments such as residential homes and office buildings. Therefore, PIR-based localization approaches can be classified according to sensor deployment, including floor-mounted, wall-mounted, and ceiling-mounted.

Hao et al. [6] developed a PIR sensor module with a modulated FOV for multi-person tracking for the floor-mounted category. The design of a modulated FOV enables the detection of multiple moving persons and facilitates data association. Kalman, HMM, and Gaussian particle filters track two targets in a 9 m × 9 m room. They achieve a distance error of 0.5 m for two targets. Yang and Zhang [8] arranged six sensors to form 12 overlapped detection zones around a sensor node. Each detection area was assigned a coding scheme to indicate a detection line. They proposed the credit-based method to filter out false measurement points, i.e., intersection points of activated detection lines. They set up a 10 m × 10 m area and deployed nine sensor nodes uniformly. The work used a Kalman filter to track a target with an average distance error of 0.42 m for two persons.

Kemper and Hauschildt [32] attached four thermopile sensors on the walls of a 4.9 m × 6.2 m area for the wall-mounted category. A Probability Hypothesis Density (PHD) filter was adopted to estimate the location of multiple targets. They achieved a mean distance error of 0.3 m for 2–3 persons. Liu et al. [33] proposed a method based on azimuth change measurements and particle filtering to localize multiple persons. In their experiment, four PIR sensors were installed in an 8 m × 8 m area. The localization results for two persons were around 0.8–0.9 m for a distance error. Another work proposed LOCI sensor modules which were developed by Narayana et al. [34]. The module contains one thermopile and one PIR sensor. Thermopiles provide location information across the FoV cone axis, while a PIR sensor estimates the location between a person and itself. K nearest neighbor (KNN) was trained to estimate a person’s possible location. In their experiment, only one sensor was mounted on a wall at a height of 1.2 m to monitor a 9 m × 8 m area, and their results showed that 82 of the distance errors were less than 0.88 m for 2–3 persons.

For the ceiling-mounted category, Tao et al. [11] deployed a total of 43 ceiling-based PIR sensors to localize multiple persons in an office room with a size of 15.0 m × 8.5 m. They integrated knowledge such as desk locations and moving directions to improve location estimation. The results showed that around 75% of the distance error was within 0.5 m. Wu et al. [35] developed the rotationally shuttered PIR (Ro-PIR) sensor. The rotating shutter allows the developed sensor node to detect a stationary person. In their experiments, each PIR sensor was attached to a 2.8 m high ceiling to monitor a circle area with a radius of 2 m. Machine learning algorithms such as KNN and SVM were employed to localize individuals and achieve a distance error within 0.44 m.

Yang et al. [36] proposed DeepPIRATES, a PIR-based indoor localization method that can be directly utilized in various deployment scenarios without retraining. DeepPIRATES integrates deep learning with a geometric model, estimating azimuth changes to infer positions, reducing the need for retraining and achieving a similar accuracy range (0.37–0.68 m) with a lower deployment density (0.08 sensors/m² vs. 0.34 sensors/m² in traditional methods). Chen et al. [37] proposed PIRILS, a deep learning-enhanced pyroelectric infrared (PIR) sensor-based system for device-free multi-target indoor localization. The system leverages overlapping fields of view (FOVs) from ceiling-mounted PIR sensors, combined with an Artificial Neural Network (ANN) using Long Short-Term Memory (LSTM) to improve tracking accuracy. A permutation invariance strategy was integrated to resolve label ambiguities in multi-target detection, and a Shuffle Sampling (SS) data augmentation technique was introduced to enhance training diversity and reduce overfitting. Experimental validation showed mean localization errors of 0.67 m, demonstrating improved stability, reliability, and response time compared to existing methods.

## 3. Methodology

This section presents the methodology of the proposed deep learning-based PIR localization for multiple persons. First, an overview of the localization scheme for multiple persons is introduced. After that, an underlying technique for data separation will be discussed. Finally, the deep CNN-LSTM model for the localization of multiple persons will be studied, and the proposed method was utilized to improve the localization accuracy.

### 3.1. Overview of Localization Method for Multiple Persons

**Figure 1:**
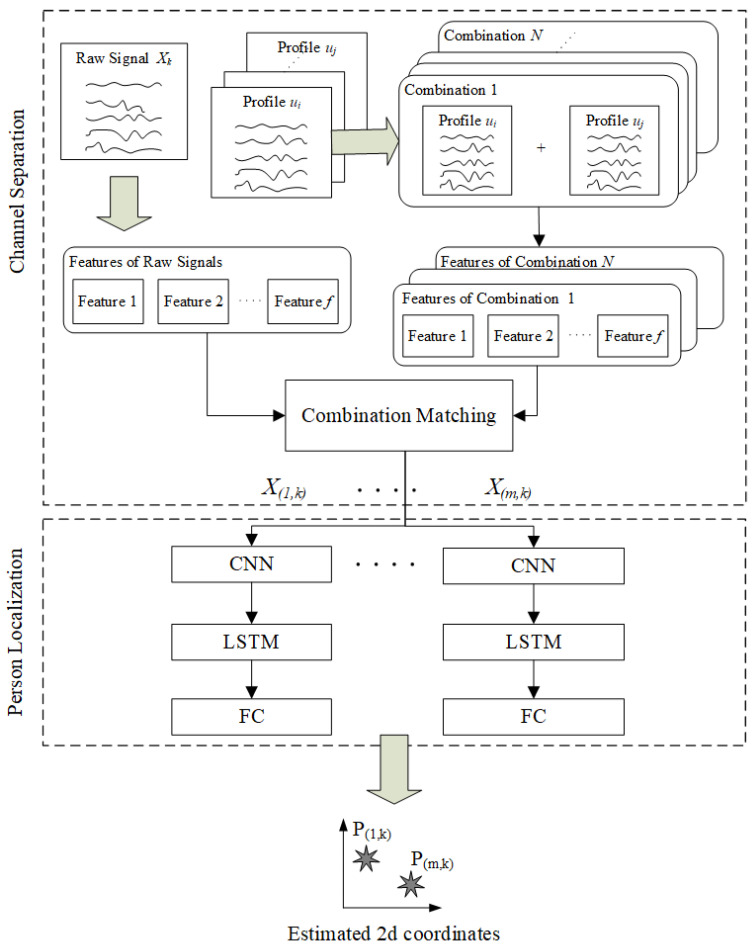
Our method for PIR-based indoor localization for multiple persons.

The proposed localization method for multiple persons consists of two main steps: channel separation and person localization. As shown in Figure 1, the method was inspired by PIRNet [38]. A monitored area *A* consists of *g* grids, and each grid consists of *c* cells. Once persons’ movements are detected by a threshold method, and raw PIR output $X_{k}$ is generated at time step *k*. The first step aims to separate a raw PIR output into *m* single-person inputs according to *m* persons. In [36], the PIR outputs of a single person were summed to approximate the outputs of multiple persons. Existing profile sequences *U* were collected from a single person, and they can be segmented into smaller profile sequences $u_{i}$, where *i* is a cell index. Then, the smaller sequences can be combined to generate *N* combinations. The raw output $X_{k}$ is compared to these combinations to find the closest match. As a result, the matched combination can help us divide the input $X_{k}$ into a set of single-person inputs $\left[X_{1,k}\ldots X_{m,k}\right]$ for the next step.

In the second step, the extracted inputs are fed to a trained deep learning model to estimate the locations of multiple persons, as shown in Figure 1. The upper CNN level focuses on extracting features. Then, an LSTM level receives the extracted features from the upper layer and learns the time dependency between extracted features. Lastly, the fully connected layer is used to output the estimated coordinates for *m* persons. In addition, multiple models can be trained and combined with their results to improve the localization accuracy for multiple persons. The details for each step will be further discussed in the following section.

### 3.2. Channel Separation

**Figure 2:**
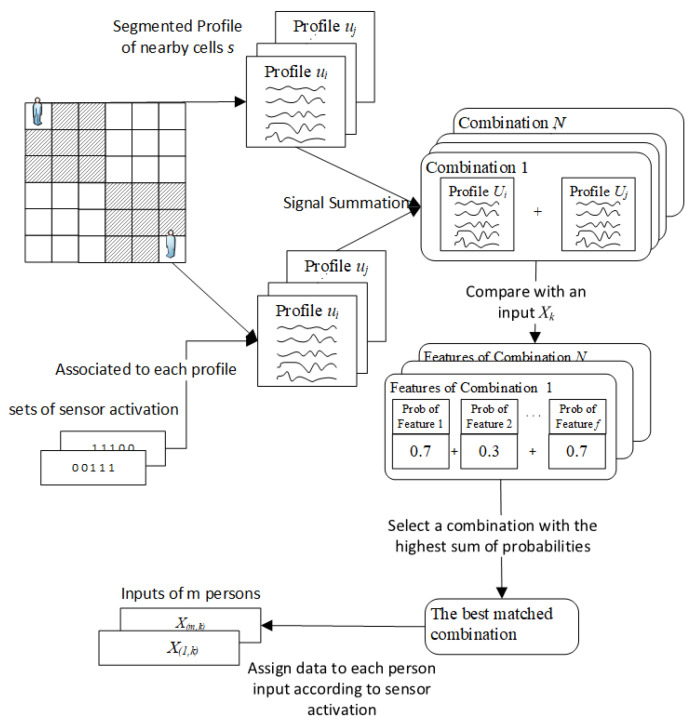
Process of channel separation in our proposed PIR-based indoor localization method.

Once persons’ movements are detected, a PIR node generates and sends raw analogue signals that can be represented as a 2D matrix with dimensions C×T, where *C* stands for a channel, and *T* is the number of samples. In this study, each output has five channels and contains 60 time steps. The raw signal $X_{k}$ is used as an input for the location estimation. The signal may contain information generated by multiple persons. For each channel, a signal needs to be analyzed to determine whether it is generated and for whom. Thus, the goal of this step is to divide the input into a set of inputs for *m* persons. When a person walks across different cells in a monitored area, distinct patterns of raw PIR signals are generated from different cells. Signal patterns can also indicate which sensors in the node are activated for each cell. Sensor activation is the information that can be utilized to separate the input data for each person. Motivated by [36], the PIR outputs of a single person are summed to approximate the outputs of multiple persons. Thus, combining profile signals from different cells will allow for the estimation of the input signals. Then, a combination can be found for a close match. As a result, the profile signals that contribute to the combination can indicate a set of sensors that each person activates. The process of this step is illustrated in Figure 2.

Next, segmenting PIR profile sequences into smaller profile sequences according to cells is studied in the monitored area. There are a number of segmented profile signals according to the total number of cells. Each segmented profile is associated with a particular set of activated sensors. Assume the number of persons and their starting locations are available. Sets of segmented profiles are selected from their nearby cells for each time step, only because computational efficiency can be considerably affected if too many profiles are selected to generate the combinations. Generally, a standard walking speed is around 1.2–1.3 m per second, and the size of a cell is 0.5 m × 0.5 m. A person can walk across two cells at a maximum of one time step. Thus, two adjacent cells surrounding a person’s location are chosen. Next, signal combinations are generated using the chosen sets of segmented profiles, and the total number of combinations *N* is calculated as follows:

(1) $$N=s_{1}\;\times \;s_{2}\;\times \;\ldots \;\times \;s_{m}$$

where $s_{i}$ denotes the number of chosen segmented profiles for the *i*th person, and *m* is the number of persons. For each combination, the selected segmented profiles are combined through a summation. Then, compare the input $X_{k}$ with all combinations to find the closely matched combination. Cross-correlation is employed, and dynamic time warping is used to compare the similarity between the input signal and profile combinations. In addition, statistical features will be used in this process, including peak-to-peak amplitude, root mean square, variance, standard deviation, mean, and mean frequency. The difference between the input and profile combinations for each feature will be calculated. Then, cross-correlation, dynamic time warping, and the difference in measurements are converted into probability values as follows:

(2) $$pb_{i,n}=\frac{v_{i,n}}{\sum_{n=1}^{N}v_{i,n}}$$

where $pb_{i,n}$ and v(i,n) denote a probability value and feature value of the *i*th feature of the *n*th combination. For each combination, the probability values will be summed up, and a profile combined with the highest probability value is selected, as shown below.

(3) $$result_{comb}=arg\;max\left(sum\left(n\right)\right)$$

where sum(n) is a sum function for probability values of the *n*th combination.

Once the possible combination is selected, the associated sets of sensor activation are utilized to generate inputs for multiple persons. *m* single-person inputs with a dimension of C×T are created according to the number of persons. For each single-person input, a signal in each channel of the input $X_{k}$ is assigned to the corresponding channel of a single-person input based on a set of activated sensors.

### 3.3. Person Localization with a Deep Learning Model

**Figure 3:**
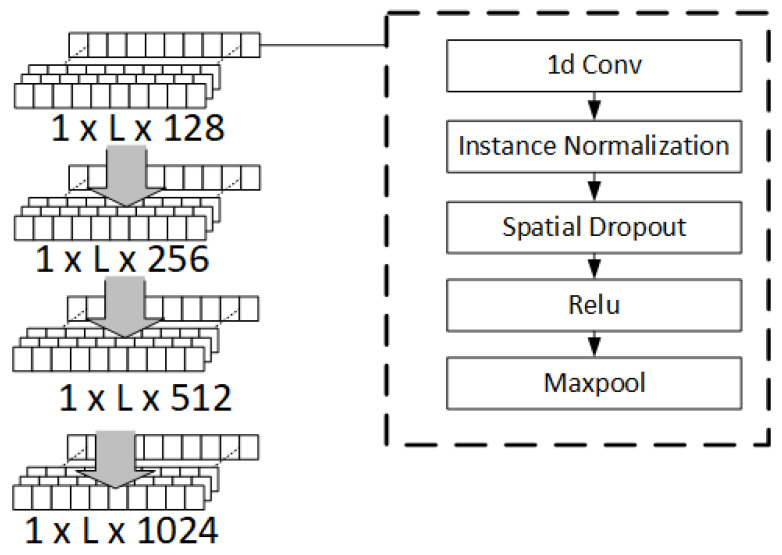
CNN layers in our model.

In this step, the channel-separated data are utilized to estimate the locations of *m* persons. A deep learning network comprising CNN and LSTM levels is proposed, as shown in Figure 3. The upper CNN level consists of 4 CNN layers with 128, 256, 512, and 1024 filters, respectively. A 1D convolutional neural network for each CNN layer is employed that performs well on time series data. The feature extraction is performed in the 1D CNN by systematically sliding a filter with its size smaller than an input across 1D sequence data to perform a dot multiplication at different parts of the sequence to discover some important features. In this study, a filter size of 3 and a stride length of 1 were set. In addition, zero padding was applied to maintain the length of the input sequence. The outputs or feature maps of the 1D CNN are normalized using an instance normalization that improves the training time and convergence of a deep learning model [39]. A spatial dropout on the normalized outputs was applied to avoid overfitting and improve generalization. This type of dropout operation is used because it was reported in [40] that a spatial dropout can improve overfitting and training time. Next, the feature maps are sent to a rectified linear action (RELU) function, which is computationally efficient and can mitigate vanishing gradient problems [41]. Finally, 1D max pooling is adopted to reduce the number of parameters and improve computational efficiency while discriminative features are maintained. In this work, the pooling window size was set to 2, and the stride length was set to 1. Zero padding was also applied for the same reason as in 1D Conv.

The lower level focuses on learning temporal patterns from time series or sequential data. LSTM is employed for this task. Compared with traditional Recurrent Neural Networks (RNN), LSTM can handle longer sequences because it utilizes the concept of input, forget, and output gates to determine which information to maintain or remove. For this reason, it is suitable to apply to a sequence of PIR outputs that is considerably long and complex.

To train the deep CNN-LSTM model, Adaptive Moment Estimation (Adam) is employed [42] to minimize a loss function to find the most optimal parameters of the deep learning model because it has excellent convergence and good computational efficiency. A loss function, namely mean square error (MSE), was chosen for the Adam optimizer. It can be calculated as follows:

(4) $$MSE=\frac{\sum_{i=1}^{j}{\left(y_{i}-\hat{y_{i}}\right)}^{2}}{j}$$

where $y_{i}$ and $\hat{y_{i}}$ are a ground-truth coordinate and a predicted coordinate, respectively. The number of samples is represented as *j*.

Multiple-person scenarios have been observed to be able to affect location estimation considerably due to the ambiguity and diversity of signal patterns. Thus, it is difficult for a single model to give an accurate result. To further improve the location estimation, 10-fold cross-validation is adopted to reduce the bias of the location estimation and avoid an overfitting issue. The training dataset can be divided into 10 slices. For every training process, 9 slices are used to train a deep learning model, and 1 slice is used for the model evaluation. As a result, 10 different models can be obtained. These models that use mean bagging are integrated and calculated as follows:

(5) $$O_{mean\;bagging}=\frac{O_{1}+O_{2}+\ldots +O_{n}}{n}$$

where $O_{i}$ is the output of the *i*th model, and *n* is the total number of models. This technique can help reduce the variance of the localization estimation. In combining these two techniques, better localization results can be achieved, as reported in [43].

## 4. Experiment Setup

In this section, we first describe our hardware and environment setup. Then, we discuss our data collection process and the scenarios used to evaluate our model.

### 4.1. Hardware and Environment Setup

**Figure 4:**
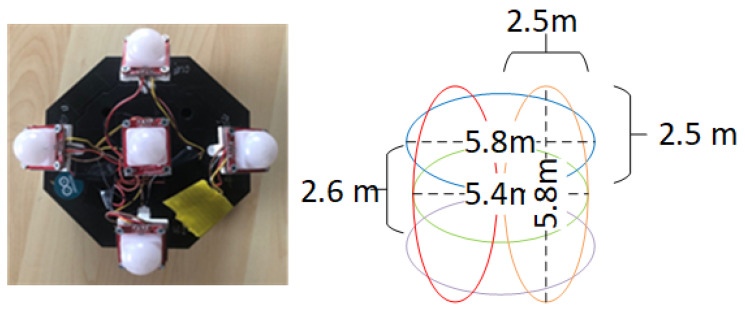
Our proposed PIR sensor node and its FOV.

A PIR sensor node was developed using five Sparkfun PIR sensors (Model SEN-13968) and an Arduino Mega 2560 microcontroller to collect training and testing data. Each PIR sensor had a Fresnel lens with a 100-degree horizontal and 60-degree vertical FOV. When detected, a movement can generate analogue outputs at a maximum of 5 volts. The FOV of each sensor was arranged as illustrated in Figure 4. The middle sensor points directly to the ground, while the surrounding sensor is angled at 30 degrees from the vertical. As a result, overlapped detection zones are formed symmetrically, which can improve the differentiation of a person’s location. The developed node was connected to a desktop computer via serial communication and transmits collected data at a rate of 60 samples per second to ensure the capture and simplification of data and time synchronization for data labeling.

**Figure 5:**
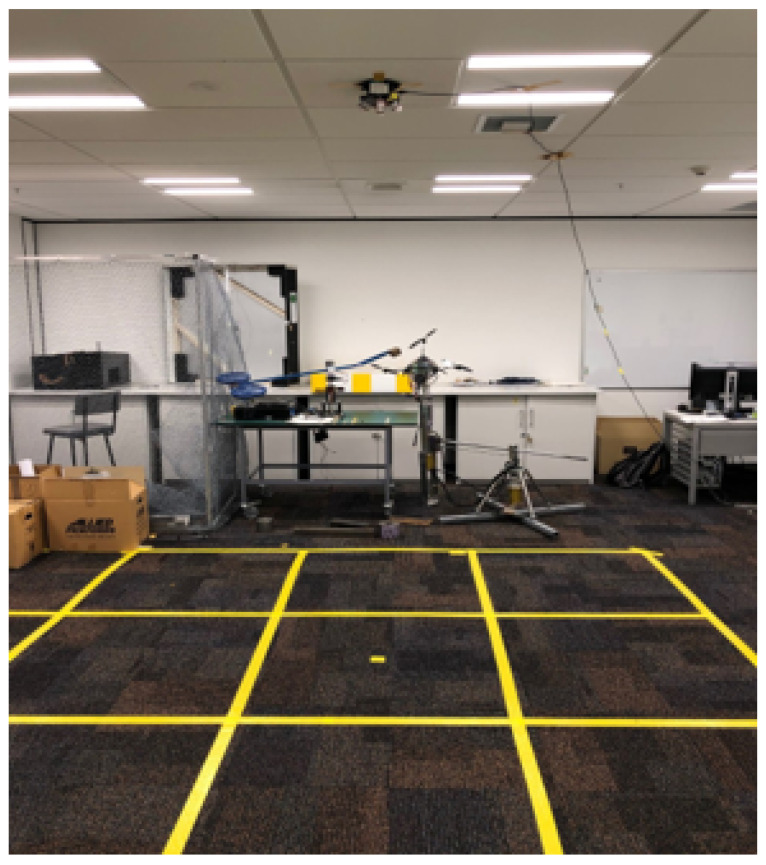
Our experiment area monitored by our ceiling-mounted PIR node.

Then, a temperature-controlled environment, such as an office room, was selected to set up a monitored area to avoid frequent changes in an environment that can affect the quality of our data collection. A square monitor area of 3 m × 3 m was prepared as shown in Figure 5. The monitored area was divided into nine grids, i.e., a grid size of 1 m^2^, and each grid could be labeled using a number from one to nine. Furthermore, each grid was further separated into four smaller cells with a size of 0.25 m^2^. Each cell was labeled with a number from one to four. The sensor node was attached to a 2.5 m high ceiling at the center of the monitored area.

### 4.2. Training and Test Data Collection

The training data were collected using a single participant in this work. A participant was instructed to walk horizontally, vertically, and diagonally across the monitored area. In this work, we assumed a person walks at a constant speed because tackling the impact of walking speed is not within the scope of this work. To maintain the speed, a metronome gave a signal to the participant for each walking step. There were six starting locations for horizontal and vertical paths, so there were 12 sequences. There were 22 sequences to be collected for a diagonal path, as the participant could walk diagonally from 11 starting locations for both the left and right sides of the area. In addition, the participant need to walk back and forth to each starting location. Therefore, 68 sequences were collected to complete one set of training data. When the same experiment was repeated 15 times, a total of 1020 sequences of PIR signals were collected as training data.

**Figure 6:**
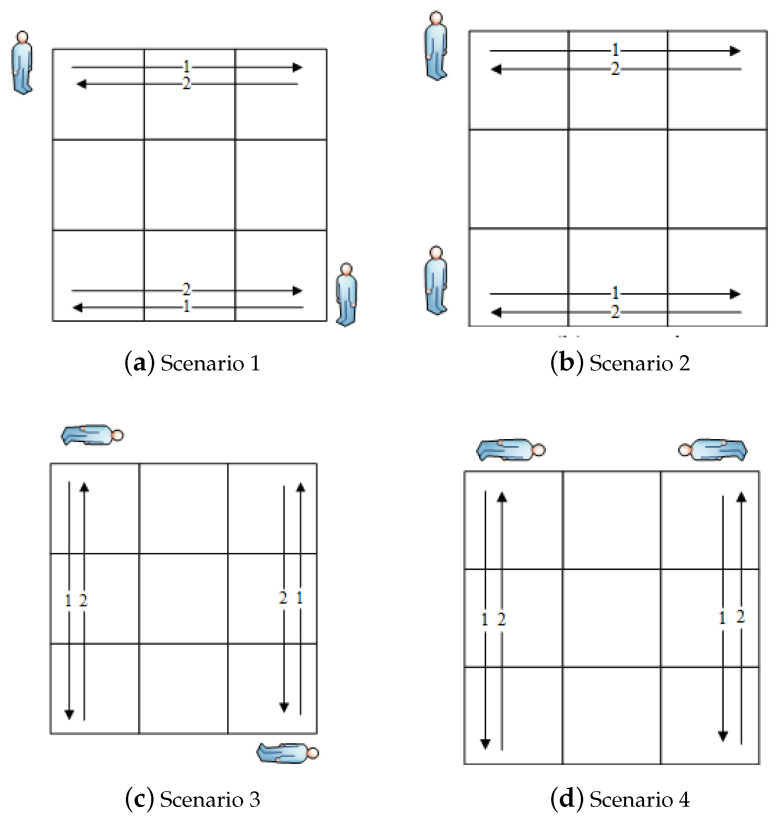
Multi-person walking scenarios.

Different walking scenarios were designed to evaluate the proposed localization method, as shown in Figure 6. Two participants participated in the testing data collection. For each scenario, both persons were instructed to walk back and forth across the monitored area at a constant speed. For Scenarios 1 and 2, two people walked horizontally across the area. However, the moving direction for each person varied between these two scenarios. Two test subjects walked in the opposite direction in Scenario 1.

In contrast, they walked in the same direction in Scenario 2. In Scenarios 3 and 4, they needed to walk vertically across the area. Similarly, the experiment also varied their walking directions in the same way as in Scenarios 1 and 2. In addition, we varied the proximity between the two participants to 2 m and 0.5 m. The settings of these scenarios helped us to analyze the overall performance of our method and factors that can affect the accuracy of localization, such as moving direction and proximity.

For training and testing data, a video camera was employed to record video footage for labeling. Then, coordinates could be extracted from this footage. After that, the deep learning model was trained and tested. The proposed methods were tested with Matlab 2020b on an i5 3.2GHz Intel quad-core CPU, RAM of 16 GB, and a GPU of NVIDIA RTX 2070 Super with 8 GB of video memory. Table 2 presents the optimal hyperparameter when used to train a deep learning model.

## 5. Results and Discussion

### 5.1. Overall Localization Accuracy

**Figure 7:**
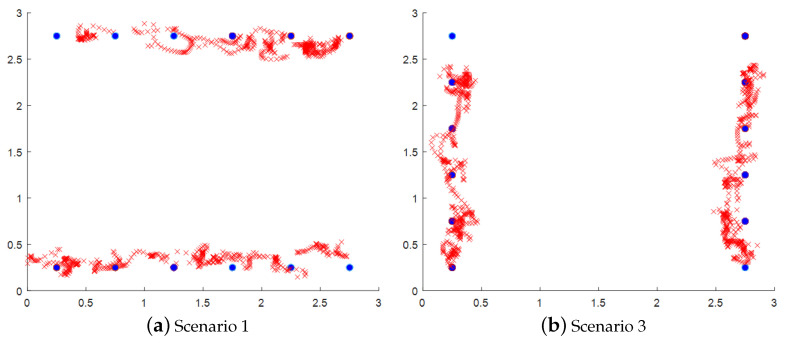
Example of PIR localization and tracking results. A red X represents the estimated location at each time step. Blue dots represent the ground-truth locations.

**Figure 8:**
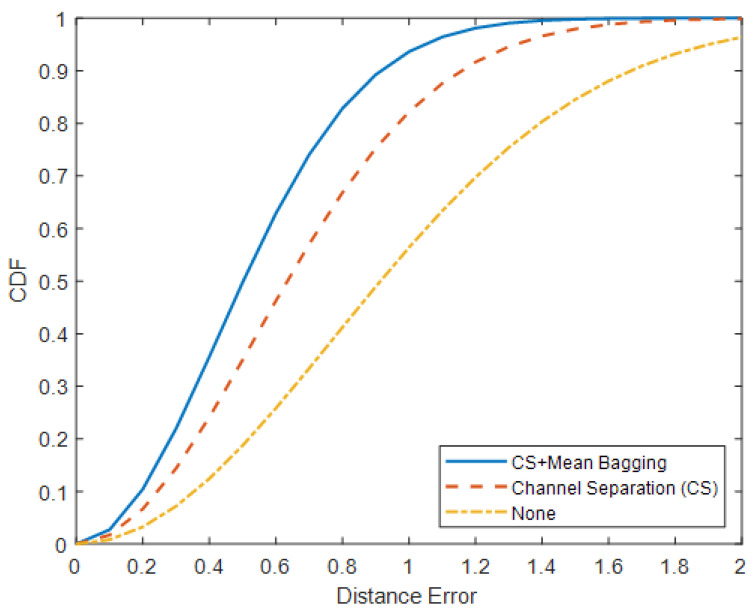
CDF of distance error for different approaches.

Visualizations of localization and tracking for two persons are shown in Figure 7, where the red paths and blue dots represent estimated locations and ground truth, respectively. It can be seen that the estimated locations for two persons follow along with the ground-truth locations as both persons walk back and forth in the monitored area. This indicates that the proposed method can localize and track two persons simultaneously. The overall accuracy of the proposed method is 0.5269 m for the distance error with a standard deviation of 0.2925 m. In addition, the CDF of the distance error shows that 70% of the distance error is less than 0.7 m, as shown in Figure 8.

In Table 3, the first row shows the location estimation results without applying any technique. As expected, the highest distance error of 1.0218 m with a standard deviation of 0.4010 m is achieved, and the CDF of the distance error shows that 70% error is less than 1.2 m because input signals for each person contain information generated by the other person. As a result, a deep learning model incorporates irrelevant information into its calculation, which results in inaccurate results. After applying the technique based on signal combination to separate input signals for both persons, the localization results improved by approximately 42%. The mean distance error is 0.6668 m, and the standard deviation is 0.3677 m. In addition, 70% of the distance error is less than 0.8 m. The best-trained model estimates the above results. Although it is the best model, a poor estimation can be made because the presence of multiple persons can obscure patterns of the raw signals. Therefore, a single model is not sufficient to estimate accurate results. After utilizing a mean bagging technique, the localization results are further improved by 23% compared to the result without the mean bagging, as shown in the third row of Table 3. Thus, it is clear that using multiple models is more favorable than a single model in multi-person scenarios, which implies multiple models can handle the ambiguity and unexpected patterns of the signals from multiple persons and integrate their results to generate better final results.

### 5.2. Impact of the Walking Direction

**Figure 9:**
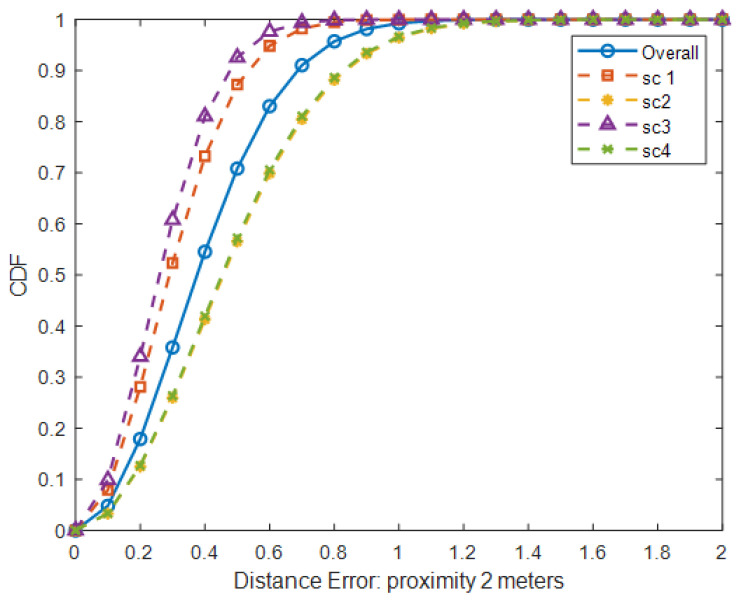
CDF of distance error for different scenarios with proximity of 2 m.

Two test subjects walk parallelly toward each other, as seen in Scenarios 1 and 3. In contrast, they walk parallelly in the same direction in Scenarios 2 and 4. The results indicate that the directions of moving persons can affect the accuracy of localization or location estimation. From Table 4 and Figure 9, the results of Scenarios 1 and 3 are more accurate than those of Scenarios 2 and 4 at 2 m. The mean distance error of Scenarios 1 and 3 is approximately 0.29 m compared to the error of 0.47 m for Scenarios 2 and 4. When two persons walk toward each other, each person is detected by a different set of PIR sensors at each time step, i.e., at least two distinct sensors are required to detect each person. Thus, patterns of raw PIR signals are less likely to be interfered with by the other person. On the other hand, two persons walking alongside each other in the same direction cause interference in patterns of PIR signals because the same set of PIR sensors detect both persons simultaneously.

### 5.3. Impact of the Proximity Between Persons

**Figure 10:**
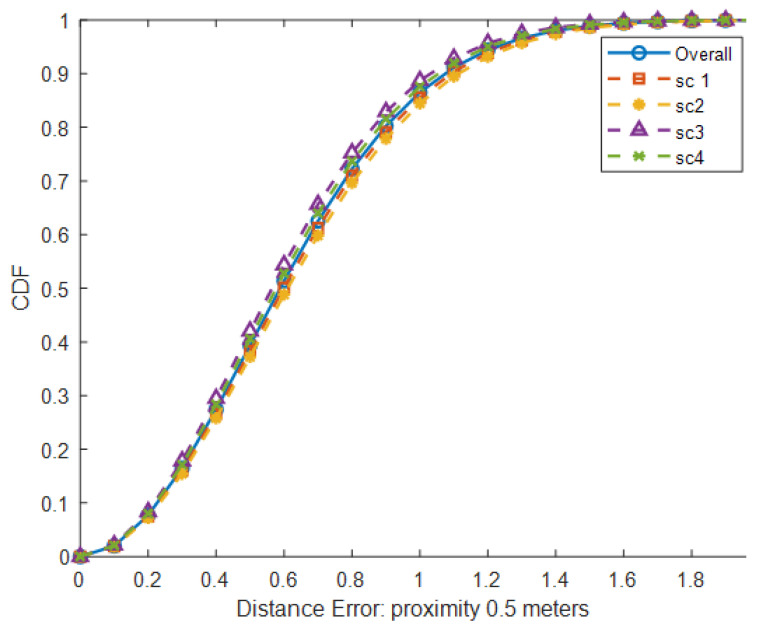
CDF of distance error for different scenarios with proximity of 0.5 m.

The proximity between persons is one factor that can affect the accuracy of localization or location estimation. In the experiment, the proximity between two persons varies between 0.5 m and 2 m. From Table 4, the overall mean distance error at 2 m proximity is around 50% lower than the overall error at 0.5 m. At 2 m away, both persons can be detected separately by different sets of PIR sensors. Patterns of raw PIR signals are reasonably clear for a deep learning model to give accurate results. On the other hand, both persons share the same PIR sensors at 0.5 m. The presence of both persons under the same sensor leads to ambiguity in PIR signal patterns, which applies to less accurate results being obtained. According to Table 4 and Figure 10, the results show that a walking direction does not affect localization accuracy significantly when the gap between two persons is 0.5 m. The movements of a person in a better position can be easily captured compared to those in the wrong position. Hence, signal patterns generated by movements at the better position become dominant in the raw PIR signal. The experiment results show that a person with a better position tends to be captured more accurately. From the above outcome, the proposed method can handle multiple persons with proximity up to 0.5 m and still obtain acceptable accuracy.

### 5.4. Impact of the Number of Models in Mean Bagging

**Figure 11:**
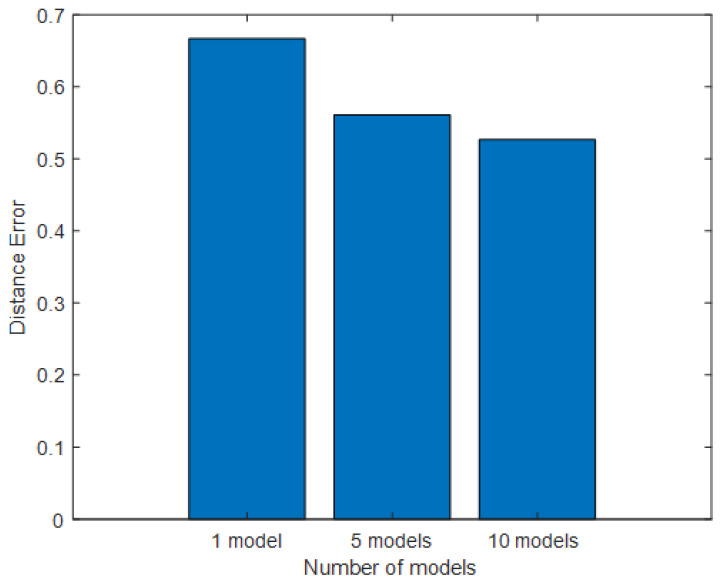
The effect of the number of models used in mean bagging.

The number of models was varied to see the effect on accuracy. The result shows that increasing the number of models can gradually improve location estimation accuracy. A single model can provide reasonably accurate estimations for a single person, but its performance is affected considerably in the presence of multiple persons. The experiment shows that a single model performs inconsistently from scenario to scenario. As a result, a single model produces the highest distance error, as shown in Figure 11. When the number of models is increased to five, it can be seen that the mean distance error is reduced by 17%. It can be observed from the experiment that five models can improve the results of scenarios in that a single model performs poorly. Using 10 models, the results can be improved by another 6%. According to the above results, an increase in the number of models in mean bagging can further reduce the variance in the location estimation. As a result, better localization results can be obtained compared to the estimation made by a single model.

### 5.5. Comparison with the Baseline Methods

In this section, the performance of the proposed deep CNN-LSTM model is compared with that of other models, including CNN-BiLSTM, CNN-GRU, and Support Vector Regression (SVR). The results of the different methods are shown in Table 5. CNN-LSTM achieves a distance error of 0.5269 m, which is the best accuracy compared with that of the other methods. CNN-BiLSTM can estimate the locations of two persons with a distance error of 0.5734 m, which is around 8.5% higher than that of CNN-LSTM. CNN-GRU obtains a distance error of 0.7752 m and performs worse than CNN-LSTM and CNN-BiLSTM. Due to the lack of a cell memory unit, GRU cannot capture the complex time dependency of PIR analogue signals very well, unlike the other RNN methods. SVR achieves a distance error of 0.8370 m for the location estimation of two persons. The overall accuracy of SVR is 45%, 37%, and 7.6%, lower than that of CNN-LSTM, CNN-BiLSTM, and CNN-GRU, respectively. SVR relies on handcrafted features to estimate locations compared to deep learning methods. Two people can be detected by the same PIR sensor simultaneously, thus introducing unexpected patterns into PIR analogue outputs. The handcrafted features may be ambiguous and are not discriminative enough for SVR to achieve accurate results. In addition, SVR cannot capture the complex time dependency of PIR signals. As a result, SVR achieves an accuracy lower than that of the deep learning models. When the gap between two persons is at 2 m, CNN-LSTM and CNN-BiLSTM can obtain a distance error of approximately 0.4 m.

In contrast, both CNN-GRU and SVR obtain worse results even when two persons stay 2 m away from each other. When a gap is closer to 0.5 m, all the methods are affected considerably for the same reasons as discussed previously in Subsection C. The proposed CNN-LSTM obtains a distance error of 0.6481 m, which is 9.7% more accurate than CNN-BiLSTM, 18% more accurate than CNN-GRU, and 35% more accurate than SVR.

Furthermore, we discuss the comparison of our method and the other two closely related works based on the PIR-based approach, DeepPIRATES [36] and PIRILS [37]. PIRILS, built based on an LSTM-based ANN with permutation-invariant learning, achieved a 0.68 m localization error in a 3.3 m × 3.3 m environment. DeepPIRATES (Hybrid Deep Learning + Geometric Model) can achieve a mean error between 0.56 and 0.71 m depending on the deployment. Compared with them, our method can achieve the lowest mean error of 0.55 m in a 3 m × 3 m environment.

## 6. Conclusions and Future Work

This paper explores a deep learning approach for device-free multi-person localization using PIR analogue signals. Based on the assumption of a constant walking speed, our proposed method can separate raw PIR outputs generated by multiple persons into a set of inputs according to the number of persons. The strategy of integrating multiple models using the mean bagging technique in our work can improve the localization results for multiple people. A set of experiments were conducted with two participants to evaluate our method, and the results show that they can achieve a mean distance error of 0.55 m, and 80% of distance errors were within 0.8 m.

However, there are some areas of improvement in our work. Although the locations of two persons with a gap of 0.5 m can be estimated, it is still an issue because the location estimation of one person is much more accurate than the other due to the ambiguity of PIR analogue signals. In future work, this issue can be studied to improve the accuracy of our multi-person localization scheme. Moreover, data collection was conducted in a temperature-controlled room. If there are changes in a room’s temperature, the accuracy of our localization method can be affected. Other factors, such as walk speed, can also affect the accuracy because our trained deep learning model does not account for these factors. The above issues could be addressed in future work. In addition, the experiment was conducted only with two people in a small-scale open area. The performance of the proposed method in real-life locations such as residential homes or apartments is still questionable. Thus, experiments could be extended to different locations. 

## Figures and Tables

**Table 1:** Comparison of existing technologies and the proposed method.

Technology	Advantages	Shortcomings	Proposed Method Improvements
**RF-Based (WiFi, RFID, UWB, mmWave)**	Long-range detection, obstacle penetration, high accuracy in ideal conditions	Susceptible to electromagnetic interference, high power consumption, high hardware cost, privacy concerns	Uses PIR sensors, which are low-cost, low-power, and privacy-preserving
**Vision-Based (Cameras, Thermal Imaging)**	High-resolution localization, useful in various lighting conditions	Privacy concerns, high processing requirements, limited in occluded environments	PIR sensors operate in any lighting condition and do not require video data
**Acoustic/Vibration-Based**	Can work in various environments without visual input	Requires specialized infrastructure, limited range, sensitive to background noise	PIR sensors are unaffected by noise, making them more versatile in indoor environments
**Existing PIR-Based Localization**	Low cost, energy efficient, unaffected by electromagnetic interference	Requires dense deployment or sophisticated nodes, lacks accuracy in multi-person scenarios due to signal interference	Uses analogue PIR signals, reducing sensor deployment while enhancing accuracy
**Deep Learning-Based Localization (Prior Work)**	Can improve accuracy over traditional methods	Training requires extensive data collection, may struggle with multiple targets in close proximity	CNN-LSTM ensemble model with mean bagging enhances accuracy in multi-person scenarios

**Table 2:** CNN-LSTM training parameter settings.

Parameter	Value
Max epoch	100
Mini batch size	32
Learning rate	0.0005
Learning rate drop factor	0.1
Gradient threshold	1
Gradient threshold method	L2norm
L2 Regularization	0.0001

**Table 3:** Mean distance errors and their standard deviations for different settings.

Setting	Distance Error	
	Mean	Std
None	1.0218 m	0.4010 m
Channel Separation (CS)	0.6668 m	0.3677 m
CS+Mean Bagging	0.5269 m	0.2925 m

**Table 4:** Mean distance errors and their standard deviations for different scenarios.

Scenario	Distance Error with Different Proximity			
	2 m		0.5 m	
	Mean	Std	Mean	Std
1	0.3085 m	0.2405 m	0.6795 m	0.3723 m
2	0.4815 m	0.2809 m	0.6403 m	0.4112 m
3	0.2809 m	0.1909 m	0.6228 m	0.4117 m
4	0.4669 m	0.3265 m	0.6501 m	0.3086 m

**Table 5:** Mean distance errors for baseline deep learning and machine learning methods.

Methods	Overall Mean Distance Error	Mean Distance Error at 2 m	Mean Distance Error at 0.5 m
CNN-LSTM	0.5269 m	0.3841 m	0.6481 m
CNN-BiLSTM	0.5734 m	0.4132 m	0.7142 m
CNN-GRU	0.7752 m	0.7606 m	0.7806 m
SVR	0.8370 m	0.7725 m	0.8953 m

## Data Availability

Data available on request from the authors.
